# Study on the molecular mechanism of chlorotoxin from Buthus martensii Karsch in the inhibition of glioma angiogenesis

**DOI:** 10.1371/journal.pone.0361186

**Published:** 2026-10-29

**Authors:** Yiming Wang, Yaning Zhao, Ziyang Ren, Yiran Chen, Liangchong Chen, Tao Hu

**Affiliations:** 1 Department of Neurosurgery, Heyou Hospital, Foshan, China; 2 Department of Neurosurgery, Shanxi Provincial People’s Hospital, Taiyuan, China; 3 Department of Neurosurgery, Xi’an International Medical Center Hospital, Xian, China; 4 Department of Neurosurgery, Wenzhou Hospital of Integrated Traditional Chinese and Western Medicine affiliated to Zhejiang Chinese Medical University, Wenzhou, China; Keio University School of Medicine Graduate School of Medicine: Keio Gijuku Daigaku Igakubu Daigakuin Igaku Kenkyuka, JAPAN

## Abstract

**Background:**

Glioma is a highly malignant intracranial tumor with dismal prognosis. Tumor angiogenesis critically drives glioma growth and invasion, making vascular endothelial cells a promising therapeutic target. Buthus martensii Karsch chlorotoxin (BmK CT) presents favorable antitumor properties, whereas its regulatory role in glioma angiogenesis and the specific mechanism remain poorly defined.

**Aim:**

To investigate the regulatory role of BmK CT in glioma angiogenesis and the underlying mechanism.

**Methods:**

HUVECs were cultured alone or exposed to glioma conditioned medium prepared from primary glioma cells. Functional assays were performed to assess the impacts of BmK CT on endothelial proliferation, migration and tube formation. Quantitative proteomics and bioinformatics analysis were applied to screen differentially expressed proteins (DEPs) and explore underlying mechanisms.

**Results:**

BmK CT significantly inhibited the proliferation of HUVEC cells and reduced their migration and tube formation abilities. BmK CT reversed the promoting effect of primary glioma cells on the migration of HUVEC cells. Proteomics identified multiple DEPs. Enrichment analysis suggested that BmK CT exerts an anti-glioma angiogenesis effect through a multi-target mechanism by inhibiting pro-angiogenic pathways, blocking energy metabolism, and regulating the inflammatory microenvironment. Furthermore, expression pattern clustering analysis revealed six DEPs expression patterns. Parallel reaction monitoring (PRM) analysis supported nine candidate proteins (THBS1, THBS4, COMP, F2, A2M, C3, RIGI, EZR, and ITGA6) associated with glioma angiogenesis. Conclusion: This study demonstrated in vitro that BmK CT inhibits endothelial angiogenic behaviors relevant to glioma progression and modulates the tumor microenvironment, and identified nine candidate DEPs, providing a theoretical basis for future molecular targeted therapy strategies for glioma.

## Introduction

As the most invasive and lethal primary malignant tumor of the central nervous system, glioma—particularly glioblastoma (GBM)—accounts for approximately 45% to 50% of adult intracranial malignant tumors globally, with a 5-year survival rate still below 5% [1]. Despite continuous optimization of comprehensive treatment systems centered on maximal safe surgical resection, concurrent radiotherapy and chemotherapy, and temozolomide adjuvant chemotherapy, the highly invasive nature, significant neovascularization characteristics, and strong tumor heterogeneity of glioma consistently limit treatment efficacy [2,3]. The median survival period after diagnosis is only 12–15 months, and the postoperative recurrence rate is close to 100% [4]. Pathological angiogenesis not only provides necessary nutrients and oxygen for rapidly proliferating tumor cells but also disrupts the integrity of the blood-brain barrier and blood-tumor barrier, constructing an abnormal vascular microenvironment and an immunosuppressive tumor microenvironment (TME), thereby promoting tumor immune escape [5]. Furthermore, multiple signaling pathways activated during angiogenesis, such as the vascular endothelial growth factor (VEGF) and its receptor signaling pathway and the phosphatidylinositol 3-kinase/protein kinase B signaling pathway, can induce resistance of glioma cells to radiotherapy and chemotherapy, forming a vicious cycle of “angiogenesis–drug resistance–malignant progression” [5,6]. Based on the core role of angiogenesis, anti-angiogenic therapy has become an important strategy in glioma treatment, among which VEGF-neutralizing antibodies represented by Bevacizumab have been applied in the clinical treatment of recurrent GBM [7]. However, clinical practice has confirmed that this therapy cannot block alternative pro-angiogenic pathways activated by glioma cells, and long-term treatment easily induces endothelial-like transformation of glioma stem cells, forming non-endothelial cell-dependent angiogenesis modes such as vascular mimicry and vessel co-option, ultimately leading to drug resistance [7,8]. Studies have found that the essence of glioma angiogenesis is an abnormal “symbiotic relationship” between tumor cells and vascular endothelial cells—glioma cells induce proliferation, migration, and lumen formation of vascular endothelial cells by secreting factors such as VEGF and matrix metalloproteinases [9]; while newly formed blood vessels provide nutrients and migration pathways for glioma cells, further promoting their infiltration and metastasis [10]. Therefore, developing novel multi-target anti-angiogenic drugs to disrupt the signaling network between glioma cells and vascular endothelial cells and to target the crosstalk mechanisms between them is expected to become a key breakthrough in overcoming the bottleneck of anti-angiogenic therapy.

Natural bioactive peptides have gradually become a focus of intense research interest in glioma treatment due to their advantages such as strong target specificity, favorable safety profiles, and multi-target synergistic effects. Among them, the anti-tumor effects of Buthus martensii Karsch chlorotoxin (BmK CT) have attracted significant attention [11]. BmK CT belongs to the chlorotoxin family of small peptides and comprises only 36 amino acid residues, including eight cysteines that form four intramolecular disulfide bonds, a scaffold that confers high structural stability in complex physiological environments. Owing to this compact and rigid framework, chlorotoxin-family peptides retain their bioactive conformation and are resistant to proteolytic degradation. The natural biological function of this peptide family is to selectively bind to chloride channels on the cell membrane, a property that underlies their high targeting specificity for glioma cells. BmK CT was originally isolated and purified from the venom of Buthus martensii Karsch. Its core structure contains functional domains that specifically bind to chloride ion channels, exhibiting high targeting specificity for glioma cells [12]. Previous studies have confirmed that BmK CT can inhibit the migration, invasion, and matrix degradation of tumor cells by blocking chloride ion channels in glioma cells and suppressing the expression and activity of matrix metalloproteinase 2/9. Simultaneously, BmK CT can induce cell cycle arrest in the G0/G1 phase, promote apoptosis, and show no significant toxicity to normal neural cells [12,13]. However, current research on BmK CT mostly focuses on its direct effects on glioma cells. It remains incompletely understood whether BmK CT can directly regulate the angiogenic potential of vascular endothelial cells within the glioma microenvironment, reverse glioma-induced pro-angiogenic responses in endothelial cells, and what underlying molecular mechanisms govern these processes. This constitutes the core scientific question to be addressed in this study. Besides directly suppressing glioma invasion and migration, BmK CT also serves as a targeting carrier for glioma theranostics. For instance, its ^131^I-labeled conjugate (^131^I-BmK CT) enables SPECT imaging of gliomas while inhibiting metastasis [14]; when attached to ^131^I-labeled dendrimers (^131^I-G5.NHAc-HPAO-(PEG-BmK CT)-(mPEG)), it achieves tumor-targeted imaging and radiotherapy; and through a recombinant plasmid (pEGFP-N1-BmK CT), it mediates gene delivery that attenuates glioma migration [15,16]. Furthermore, BmK CT has spurred the screening of other chlorotoxin-like peptides, thereby expanding the repertoire of anti-glioma peptide drug candidates [17].

This study systematically investigates the anti-angiogenic effects and mechanisms of BmK CT in glioma. First, through in vitro functional experiments, the direct effects of BmK CT on the proliferation, migration, and tube formation capacity of human umbilical vein endothelial cells (HUVECs) were assessed to elucidate its inhibitory impact on endothelial cell angiogenic activity. To mimic the pathological microenvironment of clinical glioma, a glioma conditioned-medium (GCM) exposure model, in which HUVECs were exposed to conditioned medium from primary glioma cells, was established to explore whether BmK CT can reverse the pro-angiogenic phenotype induced in endothelial cells by glioma-derived paracrine factors, thereby evaluating its therapeutic potential in the pathological microenvironment. High-throughput quantitative proteomics technology was employed to unbiasedly map the protein expression profiles of HUVECs treated with BmK CT, screening for differentially expressed proteins (DEPs). By performing Gene Ontology (GO) functional enrichment, Kyoto Encyclopedia of Genes and Genomes (KEGG) pathway analysis, and Gene Set Enrichment Analysis (GSEA), the core biological pathways and molecular modules regulated by BmK CT were identified. To ensure the reliability of the research results, parallel reaction monitoring (PRM) technology was used to validate the core roles of target proteins such as coagulation factor II (F2), α2-macroglobulin (A2M), cartilage oligomeric matrix protein (COMP), thrombospondin 1/4 (THBS1/4), and integrin α6 (ITGA6) in inflammation and vascular remodeling. Integrating the results of phenotypic assays and proteomics analysis, this study elucidated the core mechanisms of BmK CT’s anti-angiogenic effects in glioma. These findings provide new insights into the molecular pharmacology of scorpion venom peptides. Subsequent studies could further validate the anti-angiogenic efficacy of BmK CT through in vivo animal models, explore its synergistic effects when combined with radiotherapy, chemotherapy, and immunotherapy, and provide new ideas and strategies for the clinical treatment of glioma.

## Materials and methods

### Cell culture

HUVEC (iCell-h110, iCell) were cultured in HUVEC-specific medium (iCell-h110-001b, iCell). Primary glioma cell isolation and culture: Glioma tissue was obtained from a patient who underwent surgical resection at Shanxi Provincial People’s Hospital, with no prior anti-tumor treatment, and postoperative pathology confirmed Glioblastoma WHO grade 4. The study protocol was approved by the Medical Ethics Committee of Shanxi Provincial People’s Hospital, with the ethics approval number: (2023) Provincial Medical Ethics Review No. 161 and all procedures were conducted in accordance with the Declaration of Helsinki. Written informed consent was obtained from the participant before enrollment. Fresh glioma tissue was collected under a clean bench, transferred to a petri dish containing pre-cooled normal saline, washed to remove blood cells and tissue debris, and necrotic tissue was removed. Approximately 200 mg of tumor tissue was transferred to a homogenizer, gently ground until fragmented, and sequentially treated with 1 ml PBS (#P1020, Solarbio) and 0.5 ml 0.25% trypsin (#T8150, Solarbio). The tissue on the homogenizer pestle was rinsed into the homogenizer, and gentle pipetting was performed to ensure full contact between trypsin and the tumor tissue. After digestion at room temperature for 10–20 seconds, 1 ml of DMEM medium (#11965084, Thermo Fisher) containing 10% fetal bovine serum (FBS, #10099141C, Thermo Fisher) and 1% penicillin-streptomycin (#15140122, Thermo Fisher) was added to stop digestion. The mixture was centrifuged at 900 rpm for 4 minutes at room temperature, and the supernatant was discarded. The cell pellet was resuspended in DMEM medium containing 10% FBS and 1% penicillin-streptomycin, transferred to a cell culture flask, and cultured continuously. Supernatant from well-growing primary glioma cells was collected and stored at −80°C for future use. To prepare GCM, supernatant from well-growing primary glioma cells was collected and stored at −80°C; before use, GCM was mixed with fresh HUVEC-specific medium (1:1, v/v) and applied to HUVECs. All experiments involving glioma-derived factors therefore used a glioma conditioned medium model rather than a direct co-culture system. All cells were incubated at 37°C in a 5% CO2 incubator. HUVECs were used between passages 3 and 6, and primary glioma cells between passages 2 and 4. All cell lines were routinely tested for mycoplasma contamination and were confirmed negative.

### Cell viability assay

The Cell Counting Kit-8 (CCK-8, #RC3028, ReportBio) was used to evaluate the inhibitory effect of BmK CT on cell proliferation. HUVECs were seeded in a 96-well plate at a density of 5 × 10^3 cells per well in 100 μL of culture medium (a density confirmed to fall within the linear range of the assay in preliminary experiments), and subsequently treated with varying concentrations of BmK CT (0.5, 1, 1.5, 2, and 3 μM), which was synthesized and validated by Beijing Boyang Hongye Biotechnology Co., Ltd. Following a 24-hour incubation period, 10 μL of CCK-8 solution was added to each well, and the plates were incubated for an additional 2 hours. Absorbance was measured at 450 nm using a microplate reader (#keebio-MR100, JiaPeng Technology Co., Ltd.), and cell viability was subsequently calculated.

### Cell scratch assay

HUVECs were seeded at 1 × 10^5 cells per well in a 6-well plate and cultured for 24 hours. A sterile pipette tip was used to create a scratch in the center of each well. The original medium was then removed, and the wells were washed three times with PBS to remove detached cells. The first photograph was taken at this point (marked as 0h). Subsequently, 1 mL of the corresponding medium (containing 1 μM BmK CT or vehicle) was added to each well. The cells were then cultured for an additional 24 hours before the second image was captured. Image processing and analysis were performed using ImageJ (V 1.48) software. The cell migration rate (%) was calculated using the following formula: [(Scratch area at 0 h − Scratch area at 24 h)/ Scratch area at 0 h] × 100%.

### Tube formation assay

HUVECs in the logarithmic growth phase were seeded into 6-well plates at a density of 1 × 10^6^ cells/mL. When cell confluence reached 70%, 1 μM BmK CT was added for treatment. After 48 hours, the culture medium was replaced with HUVEC-specific medium, and cells were cultured for an additional 24 hours. Pre-chilled Matrigel matrix (200 μL; #354480, Corning) was added to each well of a 24-well cell culture plate on ice and allowed to polymerize. HUVECs (1 × 10^5^ cells/well) were then seeded onto the solidified Matrigel matrix. Tube formation was observed and imaged at 6 and 10 hours post-seeding using a microscope (DMIL LED, Leica).

### Flow cytometry (FC)

HUVECs were treated with 1 μM BmK CT for 48 h, collected, and resuspended for apoptosis analysis. Add 195 μL of Annexin V-FITC binding buffer, 5 μL of Annexin V-FITC, and 10 μL of propidium iodide staining solution (#C1062L, Beyotime) sequentially to the cell suspension, and incubate the mixture in the dark at room temperature for 20 min. Perform data acquisition and analysis within 1 h of staining using a CytoFLEX LX flow cytometer (Beckman Coulter).

### Immunofluorescence (IF)

Cells were fixed with 4% paraformaldehyde (#BL539A, biosharp), blocked with 5% BSA (#SW3015, Solarbio), and incubated with the PCNA (proliferating cell nuclear antigen) antibody (#ab92552, Abcam) for 2 hours or overnight. After washing with PBS, cells were incubated with Cy3-conjugated anti-rabbit IgG antibody (#ab6939, Abcam) in the dark for 1 hour. Subsequently, cells were mounted using an anti-fade mounting medium containing DAPI (#P0131, Beyotime). After the slides had dried in the dark, images were captured using an Olympus IX51 fluorescence microscope.

### Proteomics detection

Sample Preparation: Cell pellets from each group were collected by centrifugation at 12,000 rpm. Four volumes of lysis buffer (containing 8 M Urea and 1% protease inhibitor cocktail) were added, and cells were lysed via sonication. The lysate was centrifuged at 18,000 × g at 4°C for 5 min, and the supernatant was used for total protein concentration determination via the BCA method (#P0010S, Beyotime). Trypsin Digestion: To ensure consistent protein concentration across all samples, the lysis buffer was used to adjust each sample to an equal volume. Trichloroacetic acid was added to a final concentration of 20%, followed by thorough vortexing and precipitation at 4°C for 2 h. The mixture was centrifuged at 4,500 × g for 5 min, the supernatant was removed, and the precipitate was washed 2–3 times with cold acetone. After drying, the precipitate was reconstituted with TEAB buffer at a final concentration of 200 mM. DTT was added to a final concentration of 5 mmol/L, and the mixture was incubated at 56°C for 30 min for reduction. Subsequently, IAM was added to a final concentration of 11 mM, and the mixture was incubated at room temperature in the dark for 15 min for alkylation. After reduction and alkylation, trypsin was added at a protease-to-protein ratio of 1:50, and digestion was carried out at 37°C for 16 h.

Liquid Chromatography-Tandem Mass Spectrometry (LC-MS/MS) Detection: Chromatographic Conditions: Peptides were separated using an EASY-nLC 1200 ultra-high-performance liquid chromatography system (Thermo Fisher, Germany). Mobile phase A consisted of 0.1% formic acid and 2% acetonitrile, while mobile phase B consisted of 0.1% formic acid and 90% acetonitrile. The gradient elution was as follows: 0 min, 94% A and 6% B; 22.5 min, 78% A and 22% B; 26.5 min, 66% A and 34% B; 28.5 min, 20% A and 80% B, with a flow rate of 700 nl/min. Mass Spectrometry Conditions: The separated peptides were ionized using a nano-electrospray ionization (NSI) source and then introduced into an Orbitrap Exploris 480 mass spectrometer (Thermo Fisher, Germany) for analysis. The ion source voltage was set at 2,300 V, and the FAIMS compensation voltage was −45 V. The first-level mass spectrometry scan range was 350–1400 m/z with a resolution of 60,000, while the second-level mass spectrometry scan range was set to a precursor isolation window of 1.6 m/z with a resolution of 15,000. Data were collected using a data-independent acquisition (DIA) scanning mode, which involved an initial full scan followed by scanning of multiple consecutive m/z windows. Peptides were fragmented in the HCD collision cell using 27% energy, and second-level mass spectrometry analysis was performed. To improve mass spectrometry efficiency, the AGC was set to 1E6, and the maximum injection time was limited to 22 ms.

### Statistical analysis

Proteomics data analysis: The R language (v4.2.0) TCseq package (version 1.8.0) was used to analyze changes in protein expression levels. Using all identified proteins as the background set, Fisher’s exact test was employed for Gene Ontology (GO) functional enrichment analysis, while GSEA was used for pathway enrichment analysis. Differentially expressed proteins (DEPs) were screened using the absolute value of log2 fold change (|log2FC|) ≥ 0.5 and adjusted P < 0.05 as criteria, and a volcano plot was generated. For non-targeted proteomics, DEPs were processed using MaxQuant software (v1.6.15.0) against the “Homo_sapiens_9606_SP_20230103.fasta” database. Parameters were set as follows: enzyme digestion with Trypsin/P, allowing a maximum of 2 missed cleavages, a minimum peptide length of 7 amino acids, and a maximum of 5 modifications. Mass tolerances for precursor ions were set to 20 ppm for the initial search and 4.5 ppm for the main search, while the tolerance for fragment ions was set to 20 ppm. Fixed modification was carbamidomethylation of cysteine, and variable modifications included oxidation of methionine and acetylation of the protein N-terminus. The false discovery rate for protein identification and peptide-spectrum matching was set to 1%. Skyline software (v21.2) was used for PRM targeted quantitative validation of DEPs, with parameters set as: protease Trypsin [KR/P], maximum missed cleavages of 0, peptide length of 7–25 amino acid residues, and alkylation of cysteine as a required modification. Peptides and fragment ions were rigorously filtered based on high quality and specificity, with an error tolerance of ≤ 0.02 Da, to ensure accurate and reliable PRM quantification. The complete proteomics workflow was performed as follows. Peptides were acquired in 4D-DIA mode on the Orbitrap Exploris 480 coupled with FAIMS, and three biological replicates were analyzed for each group. Raw data were searched with MaxQuant against the Homo sapiens UniProt database, and the resulting protein intensity matrix was normalized by median normalization. Missing values were imputed with the k-nearest neighbor (KNN) algorithm. Proteins quantified in at least two of three replicates per group were retained for downstream analysis. Differentially expressed proteins were defined using |log2FC| ≥ 0.5 and adjusted P < 0.05.

All measurements are expressed as mean ± standard deviation, median [p25, p75], or number (percentage). Data analysis was performed using SPSS (V.25) and GraphPad Prism (V 9.1.0). Student’s t-test and Mann-Whitney U test were used for two-group continuous data. Multiple-group comparisons of continuous variables were conducted using the Kruskal-Wallis test. For categorical variables, the Chi-square test and Fisher’s exact test were applied. A two-sided p < 0.05 was considered statistically significant.

## Results

### BmK CT Inhibits HUVEC cell migration and tube formation

BmK CT reduced HUVEC viability in a dose-dependent manner across 0.5–3 μM, and the CCK-8 assay identified 1.0 μM as the optimal inhibitory concentration (Fig 1A). The inhibitory effect strengthened with prolonged exposure (24–72 h), demonstrating an exposure-time-dependent response (Fig 1B). The tube formation assay demonstrated that BmK CT treatment markedly inhibited HUVEC tube formation compared to the control group (Fig 1C). The wound healing assay confirmed that BmK CT significantly inhibited HUVEC migration by slowing the rate of wound closure (Fig 1D). FC results indicated that BmK CT significantly induced apoptosis (Fig 1E).

**Fig 1:**
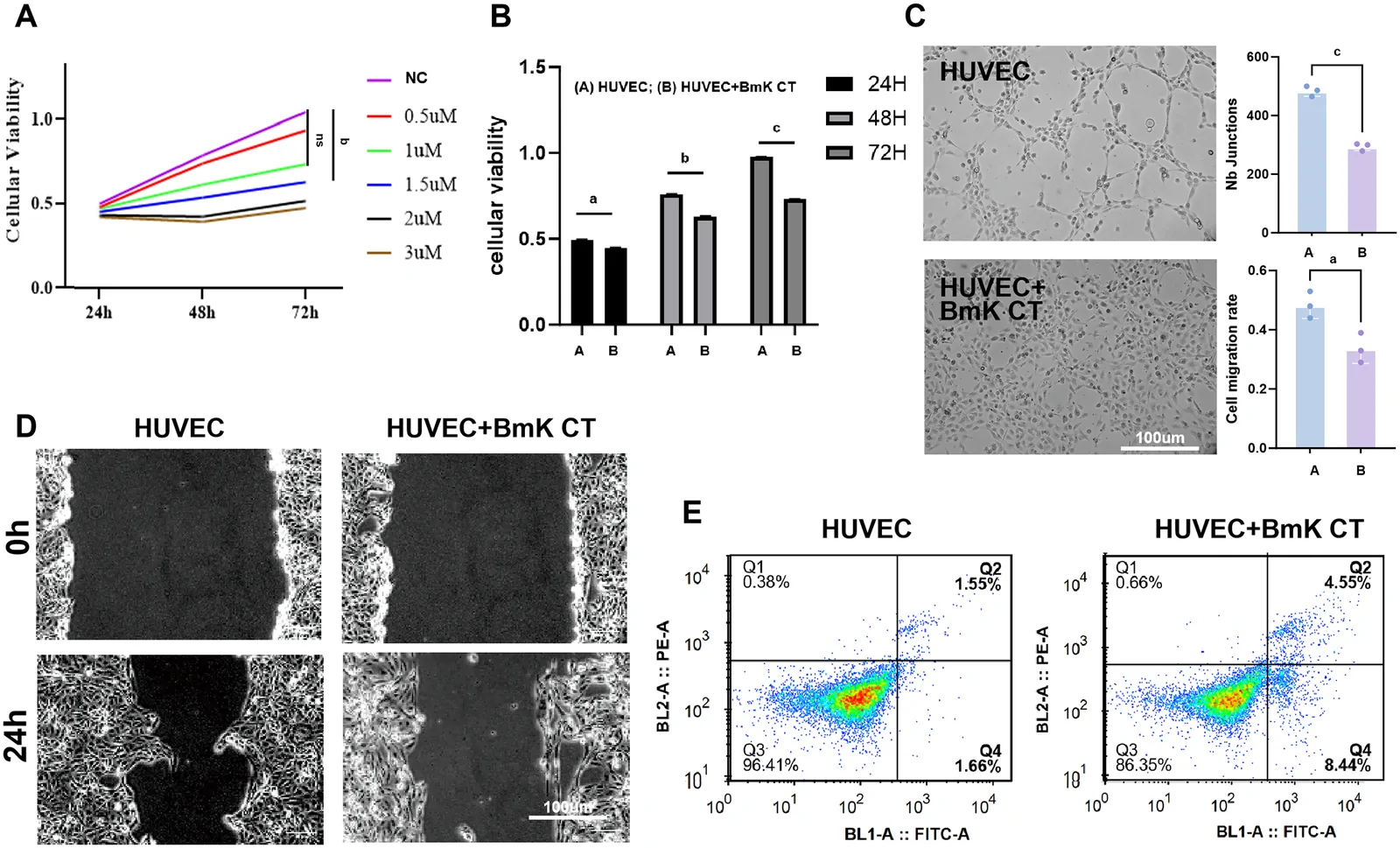
BmK CT inhibits HUVEC migration, viability, and tube formation. Note: (A) Determination of the optimal inhibitory concentration of BmK CT using the CCK-8 assay. (B) Analysis of the effect of BmK CT on the proliferation of HUVECs. (C) Representative microscopic images and quantitative analysis of tube formation. (D) Microscopic observation and quantitative analysis of the cell scratch assay. (E) Flow cytometry analysis of apoptosis in HUVEC cells after treatment in each group. ^a^P < 0.05, ^b^P < 0.01, ^c^P < 0.0001 vs control. All experiments were performed in triplicate (n = 3 independent biological replicates); data are presented as mean ± SD. Comparisons between two groups were made using Student’s t-test.

### BmK CT reverses the promoting effect of glioma primary cells on HUVEC cells

IF detection revealed that glioma primary cells widely express PCNA, indicating good proliferation status (Fig 2A). In the GCM exposure model, the CCK-8 assay confirmed that BmK CT also inhibited HUVEC cell viability, with a more significant difference at 72 h (Fig 2B). Tube formation assay results showed that the number and length of tubes formed by HUVEC cells increased in the GCM-exposure group, and BmK CT attenuated the enhanced tube-forming capacity of HUVEC cells (Figs 1C, 2C). Scratch assays confirmed that glioma primary cells promoted the migratory capacity of HUVEC cells; furthermore, compared to the GCM control, BmK CT significantly inhibited this migration in the conditioned medium group (Figs 1D, 2D). Proteomics was applied to analyze the mechanism by which BmK CT regulates the migration and proliferation ability of HUVEC cells. After BmK CT treatment of HUVEC cells, a total of 1976 DEPs were identified, including 1039 significantly upregulated DEPs and 937 significantly downregulated DEPs (Fig 2E, F). GO enrichment analysis suggested that DEPs were mainly involved in RNA processing and protein translation/processing (Fig 2G). KEGG enrichment analysis indicated that the altered proteins were primarily associated with angiogenesis-related pathways in HUVEC cells, such as the hypoxia-inducible factor-1 (HIF-1) signaling pathway, while the complement and coagulation cascades were significantly downregulated (Fig 2H). All GCM experiments included this matched vehicle-control medium, and the effects of GCM and BmK CT were evaluated relative to it.

**Fig 2:**
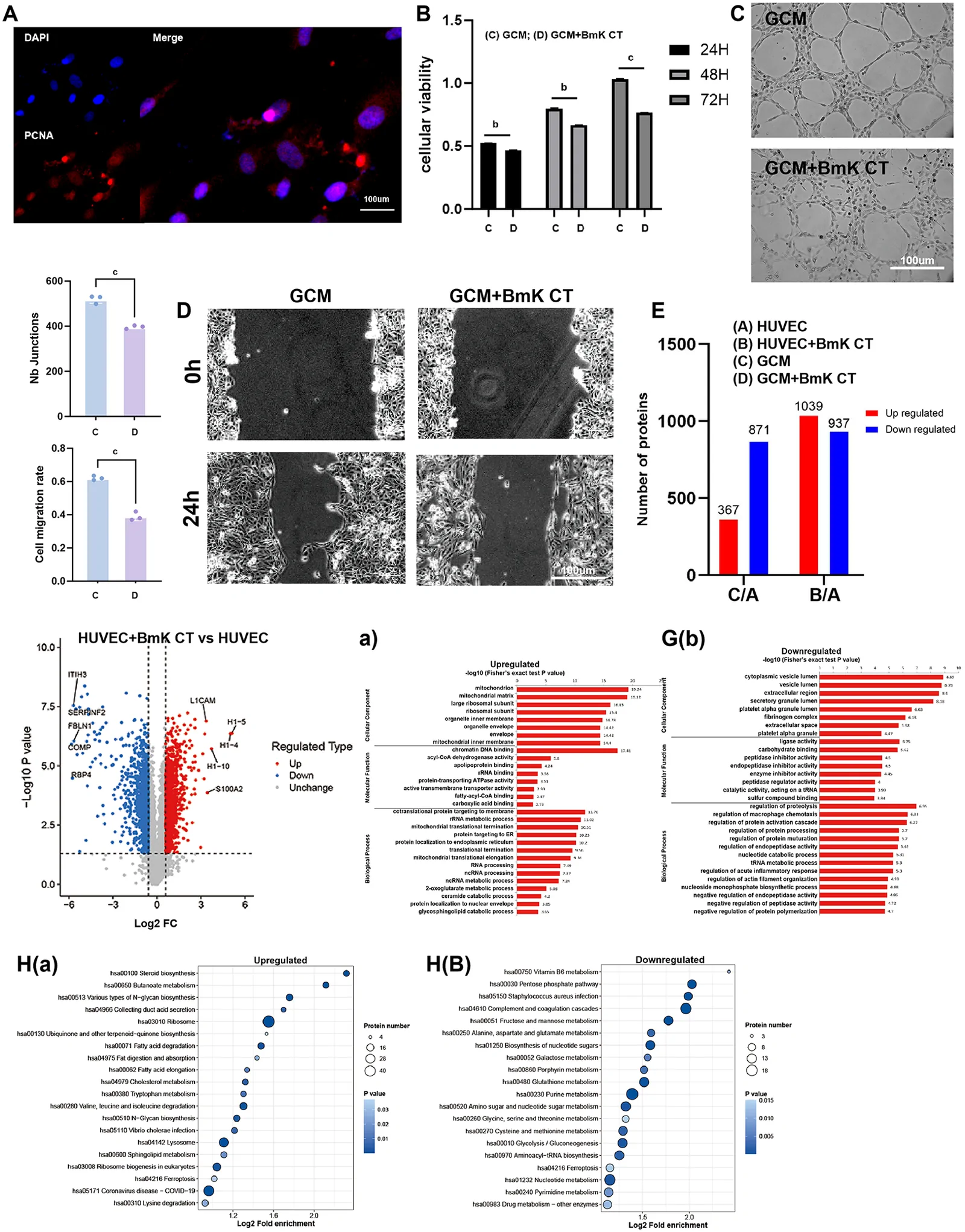
BmK CT reverses the promoting effect of glioma primary cells on HUVEC cell migration. Note: (A) Immunofluorescence (IF) staining for PCNA in glioma primary cells. (B) CCK-8 assay analysis of the effect of BmK CT on cell viability in the GCM-exposure group. (C) Microscopic observation and quantitative analysis of cell tube formation assay. (D) Representative microscopic images and quantitative analysis of the cell scratch assay. (E) Proteomic analysis of DEPs in each group. (F) Volcano plot of DEPs in HUVEC cells with or without BmK CT treatment. (G) GO enrichment analysis of DEPs in HUVEC cells with or without BmK CT treatment. (H) KEGG enrichment analysis of DEPs in HUVEC cells with or without BmK CT treatment. ^a^P < 0.01, ^b^P < 0.001, ^c^P < 0.0001 vs control. All experiments were performed in triplicate (n = 3 independent biological replicates); data are presented as mean ± SD. Comparisons between two groups were made using Student’s t-test. Proteomic analyses were based on three biological replicates per group.

### Analysis of DEPs regarding the anti-glioma angiogenesis effect of BmK CT

Compared with the HUVEC group, the GCM-exposure group exhibited a total of 1238 DEPs, including 367 significantly upregulated DEPs and 871 significantly downregulated DEPs (Figs 2E, 3A). GO enrichment analysis indicated that DEPs were mainly involved in metabolic processes, developmental processes, and transporter activity (Fig 3B). KEGG enrichment analysis showed that glioma cells primarily interfered with vascular endothelial cell metabolism and immune-related signaling pathways, such as cholesterol, glycerolipid, and tricarboxylic acid (TCA) cycle metabolism, as well as NF-κB and NOD-like receptor signaling pathways (Fig 3C).

**Fig 3:**
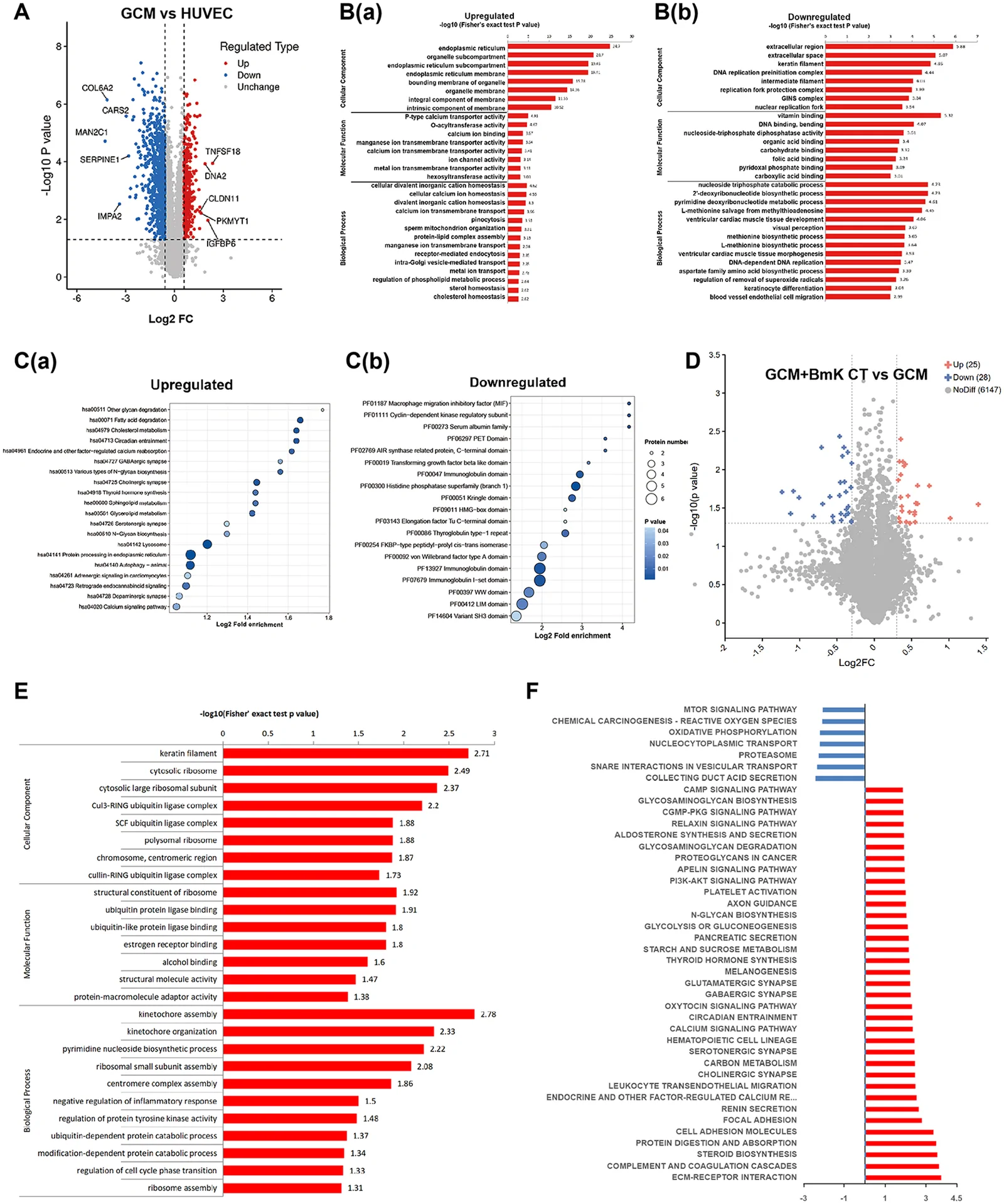
BmK CT exerts anti-glioma angiogenesis effects through multiple targets. Note: (A) Volcano plot of DEPs in HUVECs with versus without GCM exposure. (B) GO enrichment analysis of DEPs in HUVECs with versus without GCM exposure. (C) KEGG pathway enrichment analysis of DEPs identified in the GCM exposure model. (D) Volcano plot of DEPs in GCM-exposed HUVECs with versus without BmK CT treatment. (E) GO enrichment analysis of DEPs in the GCM-exposure group with versus without BmK CT treatment. (F) GSEA of DEPs in the GCM-exposure group with versus without BmK CT treatment.

A total of 6200 proteins were quantified between GCM-exposed HUVECs with and without BmK CT treatment. Among them, only 53 proteins met the criteria for differential expression, comprising 25 significantly upregulated and 28 significantly downregulated DEPs, respectively (Fig 3D, S1 File). GO functional enrichment results showed that the differential DEPs were mainly enriched in ribosome and protein translation-related pathways, the ubiquitin-proteasome system, cell cycle and proliferation-related pathways, and inflammation and signal regulation pathways (Fig 3E). GSEA results indicated that DEPs were mainly enriched in pathways related to angiogenesis and tumor progression, energy metabolism, signal transduction and cell function, and inflammation and microenvironment regulation (Fig 3F).

### Cluster analysis of DEP expression patterns

Quality control was performed using the threshold of standard deviation (SD) > 0.5 among replicate samples, ultimately including 1983 proteins for inter-group expression pattern cluster analysis. Using the fuzzy C-means clustering algorithm (number of clusters k = 6, fuzzy coefficient m = 2), these proteins were partitioned into 6 clusters with distinct expression trends. To elucidate the biological functions of proteins in each cluster, GO functional enrichment, KEGG pathway, and protein domain enrichment analyses were conducted. The results showed that in Cluster 5, proteins involved in angiogenesis-related pathways, such as the complement and coagulation cascades and extracellular matrix (ECM)-receptor interaction, were highly expressed in the HUVEC and GCM-exposure groups, with significantly downregulated expression levels after BmK CT treatment. In Cluster 6, inflammation and migration-related pathways, including the NF-κB signaling pathway, leukocyte transendothelial migration, and cell adhesion molecules (CAMs), were significantly inhibited in the BmK CT-treated GCM-exposure group (Fig 4).

**Fig 4:**
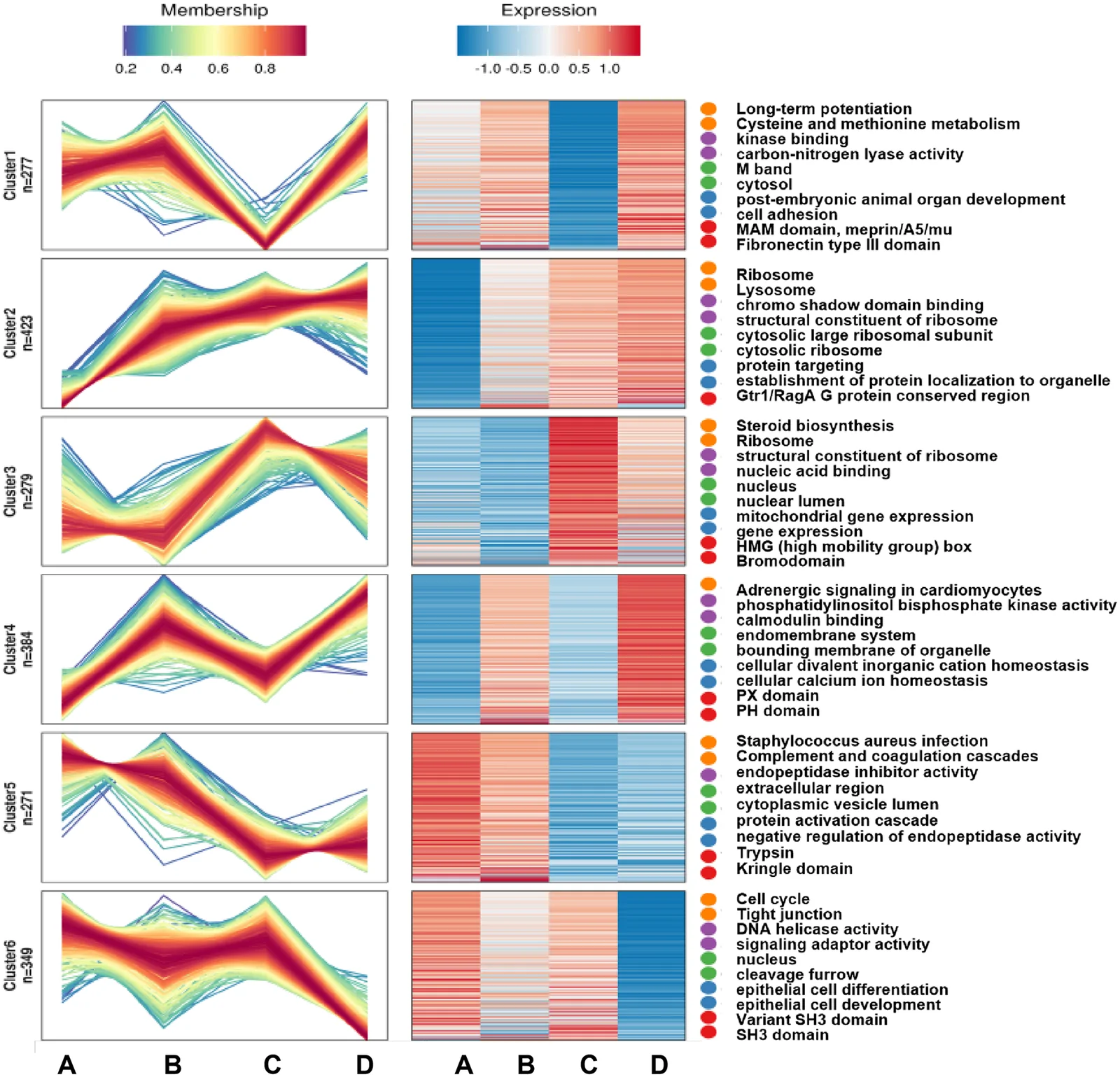
Cluster analysis of DEP expression patterns. Note: Each panel displays a line graph (left) and a heatmap (right) corresponding to a specific cluster. In the line graphs, the x-axis represents samples and the y-axis represents relative protein expression levels; each line denotes a single protein, with line color indicating the probability of that protein’s assignment to the cluster. Heatmap: The horizontal axis represents samples, the vertical axis represents different proteins, and the color intensity indicates the relative expression levels of proteins in each sample. (A) HUVEC group; (B) HUVEC + BmK CT group; (C) GCM-exposure group; (D) GCM + BmK CT group.

### Parallel reaction monitoring validation of key pathway-related proteins

The target proteins selected for validation via parallel reaction monitoring (PRM) were chosen from the aforementioned pathways to verify if their expression patterns in the GCM exposure model are consistent with previous cluster analysis results. The expression patterns of A2M and Complement C3 proteins in the complement and coagulation cascade pathway were consistent with previous cluster analysis results, whereas the direction of change for F2 differed between the 4D-DIA and PRM measurements (c/a ratio approximately 0.82 by 4D-DIA versus 1.14 by PRM), This discrepancy may reflect differences in peptide coverage, proteoform specificity, and quantification linearity between the two quantification approaches; F2 was therefore interpreted as a provisional candidate that requires further validation. The PRM analysis results of COMP and THBS1/4 in the ECM pathway also matched the cluster analysis results, further confirming their key roles in ECM remodeling. In the CAMs pathway, the expression pattern of ITGA6 protein was consistent with cluster analysis, emphasizing its importance in cell-cell interactions and adhesion processes. In the NF-κB signaling pathway, PRM analysis of the retinoic acid-inducible gene I (RIG-I) protein yielded results consistent with the cluster analysis, revealing its core role in inflammatory responses and immune regulation. In the leukocyte transendothelial migration pathway, the expression patterns of ezrin (EZR) and integrin α6 (ITGA6) were validated against the cluster analysis, highlighting their importance in immune cell migration and inflammatory processes (Table 1).

**Table 1:** PRM analysis of candidate proteins.

Protein accession	Protein description	C/A		B/A		D/C	
		4D-DIA	PRM	4D-DIA	PRM	4D-DIA	PRM
P35443	Thrombospondin-4 (THBS4)	0.69	0.71	0.01	0.07	0.05	0.22
P49747	Cartilage oligomeric matrix protein (COMP)	0.68	0.70	0.02	0.02	0.03	0.06
P07996	Thrombospondin-1 (THBS1)	0.79	0.82	0.14	0.24	0.54	0.70
P00734	Prothrombin (F2)	0.82	1.14	0.14	0.45	0.49	0.75
P01023	Alpha-2-macroglobulin (A2M)	0.73	0.76	0.03	0.10	0.30	0.58
P01024	Complement C3 (C3)	1.80	1.42	0.18	0.27	0.18	0.58
O95786	Antiviral innate immune response receptor (RIGI)	0.69	0.71	1.11	1.16	0.55	0.54
P15311	Ezrin (EZR)	1.18	1.19	1.08	1.28	0.38	0.62
P23229	Integrin alpha-6 (ITGA6)	0.84	0.86	0.84	0.86	0.23	0.56

Note: 4D-DIA: four-dimensional Data-Independent Acquisition; PRM: Parallel Reaction Monitoring. Groups were defined as follows: (A) the HUVEC group, (B) the HUVEC + BmK CT group, (C) the GCM-exposure group, and (D) the GCM + BmK CT group. Values represent the mean ratio derived from three biological replicates per group. The 4D-DIA and PRM columns show the abundance ratios between the indicated groups. Statistical significance was assessed using Student’s t-test for PRM data; proteins with adjusted P < 0.05 were considered significantly changed.

## Discussion

Glioblastoma is the most common and highly invasive primary brain tumor in adults, with an extremely poor prognosis and limited treatment options [18]. The rapid growth of this malignant tumor is highly dependent on angiogenesis, the process of forming new blood vessels from existing ones, primarily driven by pro-angiogenic factors such as VEGF-A [19]. Although anti-angiogenic therapies targeting the VEGF pathway, such as Bevacizumab, have shown initial promise in glioblastoma treatment, they have failed to significantly prolong overall survival. This failure highlights the existence of compensatory angiogenic mechanisms that allow tumors to evade VEGF inhibition [20] and underscores the urgent need to explore new therapeutic targets and strategies. In this study, in vitro functional experiments first confirmed that BmK CT significantly inhibits the proliferation, migration, and tube formation abilities of HUVECs. More importantly, in a glioma conditioned-medium exposure model that partially mimics the tumor microenvironment (TME), BmK CT demonstrated a potent antagonistic effect, effectively reversing the pro-angiogenic effect of primary glioma cells on endothelial cells. This finding suggests that BmK CT may disrupt the signaling crosstalk between glioma cells and endothelial cells, thereby breaking the vascular niche that supports tumor progression. Further exploratory proteomic analysis suggested that the anti-angiogenic effects of BmK CT are associated with the inhibition of pro-angiogenic pathways, interference with energy metabolism, and regulation of the inflammatory microenvironment; these proteomics-derived associations are hypothesis-generating and remain to be functionally validated.

In the context of pro-angiogenic signaling pathways, BmK CT treatment significantly downregulated the complement and coagulation cascade pathways in HUVECs. PRM analysis supported the decreased expression of A2M and Complement C3, whereas F2 yielded discordant results between 4D-DIA and PRM and was therefore interpreted cautiously (see PRM validation section). These proteins are not only core mediators of the inflammatory response but also directly participate in regulating vascular permeability and endothelial cell function; their downregulation may directly weaken the inflammatory signals that promote angiogenesis in the TME [21–23]. Hypoxia is a core feature of the glioma microenvironment, driving the expression of pro-angiogenic factors through the HIF-1 signaling pathway [24]. Enrichment analysis in this study suggested that BmK CT treatment is associated with downregulation of the HIF-1 signaling pathway. These findings suggest that BmK CT not only directly inhibits endothelial cell function but may also compromise cellular adaptation to the hypoxic environment. Combined with the observed alterations in RNA processing and protein translation processes from GO enrichment analysis, we speculate that BmK CT may interfere with the biosynthesis of pro-angiogenic molecules at the transcriptional or translational level, thereby producing a sustained inhibitory effect. Furthermore, the expression of COMP and THBS1/4 in the ECM-receptor interaction pathway was also inhibited, which play a crucial role in supporting the endothelial cell scaffold and transmitting survival signals [25]. GO enrichment analysis showed that DEPs were enriched in the ubiquitin-proteasome system and ribosome-related pathways. This suggests that BmK CT may disrupt protein homeostasis within endothelial cells, thereby inhibiting angiogenesis at the basal metabolic level by accelerating the degradation of pro-angiogenic proteins or suppressing their translation. Regarding energy metabolism-related signaling pathways, under GCM exposure of endothelial cells, activation of the TCA cycle may provide necessary energy and metabolic intermediates for angiogenesis. For example, α-ketoglutarate has been confirmed to directly participate in regulating the expression of angiogenesis-related genes as a co-metabolite [26]. Furthermore, in regulating pathways related to the inflammatory microenvironment, BmK CT’s inhibition of the NF-κB signaling pathway, CAMs, and the leukocyte transendothelial migration pathway was particularly significant. PRM analysis yielded changes in ITGA6, EZR, and RIGI that were consistent with the cluster analysis. Inhibition of the NF-κB pathway implies that BmK CT can downregulate vascular inflammatory responses and reduce endothelial cell activation [27]. This multi-dimensional inhibitory effect (from signal transduction to physical migration ability) allows BmK CT to maintain efficient anti-angiogenic activity even in a complex microenvironment. The NOD-like receptor signaling pathway also participates in intracellular immune monitoring and regulation of the inflammatory response, further activating the NF-κB signaling pathway by promoting the release of pro-inflammatory cytokines and chemokines, thereby influencing angiogenesis [28]. These findings systematically elucidate that BmK CT does not act on a single target but disrupts the foundation of glioma angiogenesis by interfering with a synergistic network composed of complement and coagulation cascades, ECM remodeling, energy metabolism, and inflammatory signals. This provides a new perspective for understanding its unique mechanism of action that surpasses traditional VEGF inhibitors.

At the level of protein function and cellular behavior, this study found that BmK CT interferes with fundamental biological processes such as adhesion, migration, and cytoskeletal remodeling in endothelial cells. ITGA6, a laminin receptor, mediates the adhesion of endothelial cells to the basement membrane and is crucial for vascular sprouting and stabilization [29]. Its expression was altered after BmK CT treatment, suggesting that BmK CT may disrupt the cell-matrix interactions on which endothelial cell anchoring and migration depend, thereby fundamentally inhibiting the morphogenesis of new blood vessels. EZR, as an adaptor protein linking the cell membrane to the actin cytoskeleton, plays a core role in regulating cell morphology, migration, and signal transduction [30]. Its downregulation in the leukocyte transendothelial migration pathway may directly weaken the recruitment of inflammatory cells to the tumor site and the motility of endothelial cells themselves. These results collectively indicate that the anti-angiogenic effect of BmK CT is rooted in its broad regulation of key functional genes in endothelial cells, not only inhibiting cell proliferation but also fundamentally disrupting the molecular machinery and structural basis required for complex behaviors such as migration and tube formation. This multi-level intervention may explain why BmK CT can effectively reverse the pro-migratory and pro-angiogenic effects of glioma-derived factors on HUVECs, i.e., by interfering with paracrine signals between tumor cells and endothelial cells mediated by molecules such as ITGA6 and EZR, thereby blocking the formation of a pro-angiogenic microenvironment. Compared to monoclonal antibodies primarily targeting the VEGF/VEGFR-2 axis, such as Bevacizumab, BmK CT’s action spectrum, which simultaneously targets cell adhesion, cytoskeleton, and paracrine communication, may help overcome treatment resistance caused by the activation of alternative pathways. Furthermore, this study found that BmK CT exerts a regulatory effect on the TME. The significant downregulation of Complement C3 and A2M, along with the inhibition of RIGI, a key regulator of the NF-κB pathway, strongly suggests that BmK CT functions to modulate innate immunity and the inflammatory response. After the complement system is activated in tumors, its products, such as C3a and C5a, can act as chemokines to recruit immunosuppressive cells like myeloid-derived suppressor cells and tumor-associated macrophages, which are critical contributors to the formation of the pro-angiogenic and immunosuppressive microenvironment [31]. A2M, as a broad-spectrum protease inhibitor, also participates in regulating inflammation and immune responses [32]. Therefore, BmK CT may reduce the production of pro-inflammatory factors and the recruitment of immunosuppressive cells by inhibiting the complement cascade and NF-κB signaling, thereby indirectly creating a microenvironment unfavorable for angiogenesis. This mechanism of action, which combines anti-angiogenesis with immune microenvironment regulation, has important therapeutic implications. It suggests that BmK CT can not only directly “starve” the tumor but also potentially transform the immune microenvironment from a pro-tumor state to an anti-tumor state, providing a theoretical basis for combination therapy with immune-checkpoint inhibitors and other immunotherapies. In conclusion, BmK CT exerts its inhibitory effect on glioma angiogenesis through a multidimensional network integrating inhibition of direct endothelial cell function, interference with extracellular matrix interactions, and regulation of innate immune and inflammatory pathways. The nine candidate proteins (THBS1, THBS4, COMP, F2, A2M, C3, RIGI, EZR, and ITGA6) lay a molecular foundation for the future development of multi-targeted therapeutic strategies and predictive biomarker panels for glioma angiogenesis.

Although this study systematically revealed the anti-angiogenesis mechanism of BmK CT through proteomic and functional analyses and functional validation, there are still several limitations. First, the existing data are primarily derived from in vitro cell models and conditioned-medium exposure systems, lacking direct evidence of blood-brain barrier penetration and pharmacokinetics in in vivo orthotopic glioma models. Future studies should further confirm its in vivo efficacy using orthotopic xenograft models. Second, although proteomics screening identified nine candidate proteins validated by PRM, the direct binding patterns and upstream regulatory networks involving BmK CT and these proteins (e.g., ITGA6, THBS1) have not been fully elucidated, necessitating surface plasmon resonance or co-crystallization techniques to resolve the molecular interaction details. Additionally, this study did not comprehensively investigate the heterogeneous effects of BmK CT on specific subpopulations of cells in the tumor immune microenvironment (e.g., microglia), an aspect that warrants attention in subsequent translational research. The primary glioma cells were derived from a single WHO grade 4 glioblastoma patient, limiting generalizability; validation in additional patient-derived GBM cultures and established GBM cell lines is warranted. Moreover, HUVECs are not brain microvascular endothelial cells and do not fully recapitulate the blood–brain/blood–tumor barrier, so the results should be validated in brain-derived endothelial or blood–brain barrier models. Finally, the receptor mediating chlorotoxin-family peptide effects remains controversial—proposed to be either a cell-surface glycolipid or a transmembrane protein—and no consensus exists. Clarifying this receptor will deepen our understanding of BmK CT binding and provide a theoretical basis for optimizing its imaging and targeted-therapy applications.

## Conclusion

In conclusion, this study establishes BmK CT as a highly promising multi-target candidate for glioma therapy. Its anti-angiogenic activity persisted not only on isolated endothelial cells but also in a glioma conditioned-medium model recapitulating the pro-angiogenic tumor microenvironment. Moreover, by simultaneously targeting complement and coagulation cascades, ECM remodeling, energy metabolism, and inflammatory signaling, BmK CT is less likely to be bypassed by the compensatory pathways limiting the long-term efficacy of VEGF-axis inhibitors such as bevacizumab in recurrent glioblastoma. Its favorable safety toward normal neural cells and action on the tumor endothelial compartment further support combination with standard chemoradiotherapy and immune-checkpoint blockade. Consequently, patients with high expression of the candidate proteins (e.g., THBS1/4 and ITGA6) might benefit most from BmK CT-based therapy, providing a rational basis for biomarker-guided clinical development.We not only confirmed its significant efficacy in inhibiting endothelial cell proliferation, migration, and tube formation, but also employed a systems biology approach to elucidate its complex mechanism of dismantling the tumor vascular microenvironment by remodeling the extracellular matrix, blocking energy metabolism reprogramming, and suppressing inflammatory signaling cascades. The nine candidate proteins identified, including THBS1/4 and ITGA6, constitute a unique molecular signature, providing a solid theoretical foundation for developing companion diagnostic markers and precision stratified therapy. Given its pleiotropic advantages in overcoming single-target drug resistance, BmK CT has the potential to become a novel strategy for overcoming the current limitations of glioma treatment, particularly when combined with traditional antiangiogenic drugs, potentially establishing a new paradigm for comprehensive glioma therapy. It should be emphasized that the inhibitory effects on endothelial proliferation, migration, and tube formation are directly observed phenotypes, whereas the proteomics-derived mechanistic links are exploratory associations that require functional validation.

## Supporting information

S1 File Data. (XLSX)
