# Supplementary material for: Study on the molecular mechanism of chlorotoxin from Buthus martensii Karsch in the inhibition of glioma angiogenesis
Source: PLoS One. 2026 Oct 29;21(10):e0361186. doi: 10.1371/journal.pone.0361186 (PMC13718939; doi:10.1371/journal.pone.0361186)
Supplement: Peer review history

# Peer review: Decision Letter 0

**DOI:** 10.1371/journal.pone.0361186.r001

**Authors:** Ryota Tamura

26 Aug 2026

Dear Dr. Hu,

Thank you for submitting your manuscript to PLOS One. After careful consideration, we feel that it has merit but does not fully meet PLOS One’s publication criteria as it currently stands. Therefore, we invite you to submit a revised version of the manuscript that addresses the points raised during the review process.

Please submit your revised manuscript by Sep 25 2026 11:59PM. If you will need more time than this to complete your revisions, please reply to this message or contact the journal office at plosone@plos.org. When you're ready to submit your revision, log on to https://www.editorialmanager.com/pone/ and select the 'Submissions Needing Revision' folder to locate your manuscript file.

- A letter that responds to each point raised by the academic editor and reviewer(s). You should upload this letter as a separate file labeled 'Response to Reviewers'.
- A marked-up copy of your manuscript that highlights changes made to the original version. You should upload this as a separate file labeled 'Revised Manuscript with Track Changes'.
- An unmarked version of your revised paper without tracked changes. You should upload this as a separate file labeled 'Manuscript'.

If you would like to make changes to your financial disclosure, please include your updated statement in your cover letter. Guidelines for resubmitting your figure files are available below the reviewer comments at the end of this letter.

If applicable, we recommend that you deposit your laboratory protocols in protocols.io to enhance the reproducibility of your results. Protocols.io assigns your protocol its own identifier (DOI) so that it can be cited independently in the future. For instructions see: https://journals.plos.org/plosone/s/submission-guidelines#loc-laboratory-protocols. Additionally, PLOS One offers an option for publishing peer-reviewed Lab Protocol articles, which describe protocols hosted on protocols.io. Read more information on sharing protocols at https://plos.org/protocols?utm_medium=editorial-email&utm_source=authorletters&utm_campaign=protocols.

As the corresponding author, your ORCID iD is verified in the submission system and will appear in the published article. PLOS supports the use of ORCID, and we encourage all coauthors to register for an ORCID iD and use it as well. Please encourage your coauthors to verify their ORCID iD within the submission system before final acceptance, as unverified ORCID iDs will not appear in the published article. *Only*  the individual author can complete the verification step; PLOS staff *cannot*  verify ORCID iDs on behalf of authors.

We look forward to receiving your revised manuscript.

Kind regards,

Ryota Tamura

Academic Editor

PLOS One

Journal Requirements:

When submitting your revision, we need you to address these additional requirements.

1.Please ensure that your manuscript meets PLOS ONE’s style requirements, including those for file naming. The PLOS ONE style templates can be found at https://journals.plos.org/plosone/s/file?id=wjVg/PLOSOne_formatting_sample_main_body.pd f and https://journals.plos.org/plosone/s/file?id=ba62/PLOSOne_formatting_sample_title_authors_ affiliations.pdf

2. We note that the grant information you provided in the ‘Funding Information’ and ‘Financial Disclosure’ sections do not match. When you resubmit, please ensure that you provide the correct grant numbers for the awards you received for your study in the ‘Funding Information’ section.

3. Please note that funding information should not appear in any section or other areas of your manuscript. We will only publish funding information present in the Funding Statement section of the online submission form. Please remove any funding-related text from the manuscript.

4. In the online submission form you indicate that your data is not available for proprietary reasons and have provided a contact point for accessing this data. Please note that your current contact point is a co-author on this manuscript. According to our Data Policy, the contact point must not be an author on the manuscript and must be an institutional contact, ideally not an individual. Please revise your data statement to a non-author institutional point of contact, such as a data access or ethics committee, and send this to us via return email. Please also include contact information for the third party organization, and please include the full citation of where the data can be found.

5. Please upload a new copy of Figure 1 to 4 as the detail is not clear. Please follow the link for more information: https://journals.plos.org/plosone/s/figures.

6. Please include captions for your Supporting Information files at the end of your manuscript, and update any in-text citations to match accordingly. Please see our Supporting Information guidelines for more information: http://journals.plos.org/plosone/s/supporting-information.

7. If the reviewer comments include a recommendation to cite specific previously published works, please review and evaluate these publications to determine whether they are relevant and should be cited. There is no requirement to cite these works unless the editor has indicated otherwise.

8. Please review your reference list to ensure that it is complete and correct. If you have cited papers that have been retracted, please include the rationale for doing so in the manuscript text, or remove these references and replace them with relevant current references. Any changes to the reference list should be mentioned in the rebuttal letter that accompanies your revised manuscript. If you need to cite a retracted article, indicate the article’s retracted status in the References list and also include a citation and full reference for the retraction notice.

[Note: HTML markup is below. Please do not edit.]

Reviewer's Responses to Questions

**Comments to the Author**

1. Is the manuscript technically sound, and do the data support the conclusions?

Reviewer #1: Yes

Reviewer #2: Yes

**********

2. Has the statistical analysis been performed appropriately and rigorously?

Reviewer #1: Yes

Reviewer #2: Yes

**********

3. Have the authors made all data underlying the findings in their manuscript fully available?

The PLOS Data policy

Reviewer #1: Yes

Reviewer #2: Yes

**********

4. Is the manuscript presented in an intelligible fashion and written in standard English?

Reviewer #1: Yes

Reviewer #2: Yes

**********

Reviewer #1: I would like to congratulate the authors on this well-designed and comprehensive study investigating the molecular mechanisms of Buthus martensii Karsch chlorotoxin (BmK CT) in the inhibition of glioma angiogenesis. The integration of functional phenotypic assays with high-throughput quantitative proteomics (DIA) and PRM validation provides high-quality data. The discussion is informative, and well structured and very well written. The study limitations were well-assessed. As authors mentionned, this work highlights a promising multi-target therapeutic path for glioma therapy, BmK CT while combined with traditional antiangiogenic treatment or maybe with chemotherapeutic also. I recommend a minor revisions to be addressed in methods part, some informations are missing :

The authors mention in the Abstract and Introduction that HUVECs were cultured alone or co-cultured with primary glioma cells. However, in the Materials and Methods section, they forgot to mention the co-culture part. The text only mentions that the primary glioma cell supernatant was collected and stored at -80°C. This needs to be adressed on Methods and all misssing places.

Also, the number of replicates need to be mentionned in the Methods section.

Reviewer #2: This study investigates the effects of Buthus martensii Karsch chlorotoxin on glioma associated angiogenesis using endothelial functional assays, primary glioma conditioned culture, quantitative proteomics, and PRM validation. The findings suggest that BmK CT suppresses endothelial proliferation, migration, tube formation, and regulates angiogenic, ECM, metabolic, and inflammatory pathways. The study is interesting and suitable for publication after minor revision.

Minor Comments

1. Clarify the number of independent biological replicates used for the functional assays and proteomic analysis.

2. The primary glioma cells appear to originate from a single patient. Please acknowledge this limitation and provide IDH and MGMT status, if available.

3. Since BmK CT reduces HUVEC viability and induces apoptosis, please clarify whether the observed effects on migration and tube formation are independent of reduced cell viability.

4. The discussion of ECM remodeling and invasion could be strengthened. Relevant studies on COL1A1 associated glioma invasion (Comba et al., Nature Communications), ECM organization, and oncostream dynamics would provide useful context for the roles of THBS1, THBS4, COMP, ITGA6, and EZR.

5. Better connect the endothelial findings with glioma invasion, particularly given the reported effects of BmK CT on MMP2, MMP9, adhesion, and matrix remodeling. Studies examining COL1A1, MMP associated ECM remodeling, and spatiotemporal oncostream dynamics in glioma would be particularly relevant here (Advanced Science, 2024).

6. PRM validates expression patterns but does not establish functional dependency. Please moderate the description of the identified proteins as therapeutic targets.

7. Claims regarding immune microenvironment modulation should be presented more cautiously because the current experiments do not directly evaluate immune cell function.

8. The conclusions regarding therapeutic potential should remain cautious until validated in orthotopic in vivo models.

9. Verify the number of validated DEPs and ensure consistency across the Abstract, Results, Table 1, and Discussion.

10. Figure 3 contains placeholder terms such as “specific condition,” “Condition X,” and “Condition Y.” Please replace these with the actual experimental groups.

11. Revise the Data Availability statement to comply with PLOS ONE requirements for access to the underlying experimental and proteomic data.

**********

what does this mean?). If published, this will include your full peer review and any attached files.

If you choose “no”, your identity will remain anonymous but your review may still be made public.

**Do you want your identity to be public for this peer review?**  For information about this choice, including consent withdrawal, please see our Privacy Policy

Reviewer #1: No

Reviewer #2: **Yes:**  Syed M. Faisal, Ph.D.

**********

[NOTE: If reviewer comments were submitted as an attachment file, they will be attached to this email and accessible via the submission site. Please log into your account, locate the manuscript record, and check for the action link "View Attachments". If this link does not appear, there are no attachment files.]

To ensure your figures meet our technical requirements, please review our figure guidelines: https://journals.plos.org/plosone/s/figures

You may also use PLOS’s free figure tool, NAAS, to help you prepare publication quality figures: https://journals.plos.org/plosone/s/figures#loc-tools-for-figure-preparation.

NAAS will assess whether your figures meet our technical requirements by comparing each figure against our figure specifications.

# Author comment: Author response to Decision Letter 1

**DOI:** 10.1371/journal.pone.0361186.r002

8 Sep 2026

Dear Prof. Ryota Tamura,

Academic Editor

PLOS One

Thank you very much for your decision letter and advice on our manuscript (PONE-D-26-32891) entitled “Study on the Molecular Mechanism of Chlorotoxin from Buthus martensii Karsch in the Inhibition of Glioma Angiogenesis”. We also thank the reviewers for the constructive comments and suggestions. We have revised the manuscript accordingly, and all amendments are indicated by red font in the revised manuscript. In addition, our point-by-point responses to the comments are listed below this letter.

We hope that our revised manuscript is now acceptable for publication in your journal and look forward to hearing from you soon.

With best wishes,

Yours sincerely,

Tao Hu et al.

Replies to Reviewer 1:

I would like to congratulate the authors on this well-designed and comprehensive study investigating the molecular mechanisms of Buthus martensii Karsch chlorotoxin (BmK CT) in the inhibition of glioma angiogenesis. The integration of functional phenotypic assays with high-throughput quantitative proteomics (DIA) and PRM validation provides high-quality data. The discussion is informative, and well structured and very well written. The study limitations were well-assessed. As authors mentionned, this work highlights a promising multi-target therapeutic path for glioma therapy, BmK CT while combined with traditional antiangiogenic treatment or maybe with chemotherapeutic also. I recommend a minor revisions to be addressed in methods part, some informations are missing:

The authors mention in the Abstract and Introduction that HUVECs were cultured alone or co-cultured with primary glioma cells. However, in the Materials and Methods section, they forgot to mention the co-culture part. The text only mentions that the primary glioma cell supernatant was collected and stored at -80°C. This needs to be adressed on Methods and all misssing places. Also, the number of replicates need to be mentionned in the Methods section.

Reply: We sincerely thank you for the constructive comments, which have significantly improved the clarity and reproducibility of our manuscript.

(1) We apologize for the insufficient description of the co-culture system. In the revised manuscript, we have added a complete description of the co-culture system in the "Cell culture" subsection. Specifically, primary glioma cells (passages 1–3) were seeded in the upper chamber of Transwell inserts (0.4 µm pore size polyester membrane, Corning), while HUVEC cells were cultured in the lower chamber. After 48 h of co-culture, the conditioned medium derived from glioma cells in the lower chamber was collected, centrifuged at 1,000 × g for 10 min to remove cellular debris, aliquoted, and stored at −80 °C for subsequent use.

(2) We have explicitly stated in the “Methods” subsections that all in vitro functional assays (CCK-8, Cell Scratch Assay, Tube Formation Assay, et al) and proteomic analyses were performed with three independent biological replicates. Please review again.

Replies to Reviewer 2 (Syed M. Faisal, Ph.D.):

This study investigates the effects of Buthus martensii Karsch chlorotoxin on glioma associated angiogenesis using endothelial functional assays, primary glioma conditioned culture, quantitative proteomics, and PRM validation. The findings suggest that BmK CT suppresses endothelial proliferation, migration, tube formation, and regulates angiogenic, ECM, metabolic, and inflammatory pathways. The study is interesting and suitable for publication after minor revision.

Minor Comments

1. Clarify the number of independent biological replicates used for the functional assays and proteomic analysis.

Reply: Thank you very much for helping us raise this important methodological clarification. In the revised "Methods" section, we have now explicitly stated that all functional assays (CCK-8, Cell Scratch Assay, Tube Formation Assay, et al) and proteomic analyses were performed with three independent biological replicates. Please review again.

2. The primary glioma cells appear to originate from a single patient. Please acknowledge this limitation and provide IDH and MGMT status, if available.

Reply: Thank you again for your professional suggestion. We have added the following statement in the "Cell culture" subsection: "The primary glioma cells used in this study were derived from a single surgical specimen of a patient with WHO grade 4 glioma, and were confirmed as isocitrate dehydrogenase (IDH) wild-type according to the WHO 2021 classification. The O6-methylguanine-DNA methyltransferase (MGMT) promoter methylation status was not determined." Because the primary glioma cells were obtained from a single patient, this limitation has also been explicitly addressed in the “Discussion” section. Future studies will validate the above findings using glioma cells from multiple patients with well-defined molecular subtypes. Please review again.

3. Since BmK CT reduces HUVEC viability and induces apoptosis, please clarify whether the observed effects on migration and tube formation are independent of reduced cell viability.

Reply: Thank you for pointing out this important methodological concern. First, the optimal inhibitory concentration of BmK CT was determined to be 1.0μM by the CCK-8 assay. To exclude the possibility that the inhibitory effects of BmK CT on HUVEC migration and tube formation were confounded by decreased cell viability, we further performed CCK-8 assays at the experimental endpoints (24h for the migration assay; 10h for the tube formation assay) following BmK CT (1.0μM) treatment, which confirmed that HUVEC viability was not significantly reduced (as shown in the figure below). Therefore, the observed inhibition of migration and tube formation reflects a genuine anti-angiogenic effect of BmK CT rather than a secondary consequence of cytotoxicity.

4. The discussion of ECM remodeling and invasion could be strengthened. Relevant studies on COL1A1 associated glioma invasion (Comba et al., Nature Communications), ECM organization, and oncostream dynamics would provide useful context for the roles of THBS1, THBS4, COMP, ITGA6, and EZR.

Reply: Thank you for the valuable suggestion. We have substantially expanded the relevant content in the “Discussion” section to better contextualize our proteomic findings within the framework of ECM remodeling and glioma invasion. Specifically: (i) We discussed the role of THBS1/4 as regulators of matricellular proteins in angiogenesis and glioma invasion, with references to PMID: 35750880 and PMID: 38384234; (ii) We interpreted the upregulation of COMP in the context of collagen fibril assembly and glioma ECM organization; (iii) We discussed the role of ITGA6 (α6 integrin) as a key mediator of ECM adhesion and invasion, including its interaction with MMP2/MMP9; (iv) We interpreted the downregulation of EZR from the perspective of cytoskeleton–ECM coupling and oncostream dynamics. We also cited the recently proposed spatiotemporal "oncostream" model of glioma invasion, which provides a unifying conceptual framework linking the endothelial compartment with the invasive compartment. These revisions have made the connections between our proteomic findings and the literature on glioma invasion and ECM remodeling much clearer.

5. Better connect the endothelial findings with glioma invasion, particularly given the reported effects of BmK CT on MMP2, MMP9, adhesion, and matrix remodeling. Studies examining COL1A1, MMP associated ECM remodeling, and spatiotemporal oncostream dynamics in glioma would be particularly relevant here (Advanced Science, 2024).

Reply: Thank you again for the constructive suggestion. We have revised the “Discussion” section to better integrate our findings regarding BmK CT mediated inhibition of MMP2/MMP9 and adhesion/matrix remodeling pathways with the literature on glioma invasion. Specifically, we have now explicitly established the following connections: (i) the observed inhibition of MMP2/MMP9 with the well-established roles of these matrix metalloproteinases in glioma ECM degradation and invasion; (ii) the regulation of COL1A1 and collagen-binding proteins in the context of glioma ECM remodeling; and (iii) the impact on focal adhesion/integrin signaling and glioma cell invasion. We have added the suggested citations (PMID: 35750880, PMID: 38384234) to support these mechanistic links. We look forward to your re-review.

6. PRM validates expression patterns but does not establish functional dependency. Please moderate the description of the identified proteins as therapeutic targets.

Reply: Thank you for your important advise. We acknowledge that PRM validation can only confirm expression changes at the protein level and does not establish functional causality. Accordingly, we have toned down the relevant statements in the revised manuscript. Specifically: (i) The PRM-validated proteins (THBS1/4, COMP, F2, A2M, C3, RIGI, EZR, ITGA6) are now described as "candidate" or "putative" mediators of BmK CT action, rather than "therapeutic targets"; (ii) Wherever the PRM-validated proteins are referred to, the term "target" has been replaced with "candidate mediator" or "candidate biomarker"; (iii) The final paragraph of the “Discussion” explicitly states: Before these candidate molecules can be considered genuine therapeutic targets, functional validation through gene knockdown/overexpression and rescue experiments is required. Please review again.

7. Claims regarding immune microenvironment modulation should be presented more cautiously because the current experiments do not directly evaluate immune cell function.

Reply: We thank the reviewer for raising this important caveat. We fully acknowledge that this study did not directly assess immune cell function and therefore cannot support claims that BmK CT modulates the glioma immune microenvironment. In the revised manuscript, we have made the following changes: (i) We have removed any direct claims asserting that BmK CT modulates the glioma immune microenvironment; (ii) We have revised the relevant wording in the “Abstract” and “Discussion” to state that the proteomic data are "consistent with—but not confirmatory of—a possible indirect effect on inflammatory/immune signaling, pending future validation"; (iii) We have added an explicit limitation statement: "No immune cell experiments (e.g., T cell cytotoxicity, macrophage polarization, microglial activation) were conducted in this study; therefore, immune-related inferences should be regarded as hypothesis-generating results only". We look forward to your re-review.

8. The conclusions regarding therapeutic potential should remain cautious until validated in orthotopic in vivo models.

Reply: We thank the reviewer for this important reminder. We have toned down the conclusions regarding therapeutic potential throughout the manuscript: (i) The “Abstract” has been revised to state that BmK CT "represents a promising lead compound for anti-angiogenic therapy in glioma, warranting further in vivo validation"; (ii) The “Discussion” now explicitly states: "The in vitro findings of this study should be regarded as proof-of-concept, and therapeutic potential can only be established after rigorous evaluation in orthotopic in vivo glioma models (such as patient-derived xenograft models or immunocompetent GL261 models), combined with pharmacokinetic/pharmacodynamic (PK/PD) and toxicity studies"; (iii) The “Conclusion” section ends with the following statement: "Prior to clinical translation, the anti-glioma efficacy of BmK CT must be validated in orthotopic animal models, including dose exploration, survival benefit, and safety assessment." We look forward to your re-review.

9. Verify the number of validated DEPs and ensure consistency across the Abstract, Results, Table 1, and Discussion.

Reply: We sincerely apologize for this oversight. We have carefully re-counted and cross-checked the number of DEPs across all relevant sections of the manuscript.

10. Figure 3 contains placeholder terms such as “specific condition,” “Condition X,” and “Condition Y.” Please replace these with the actual experimental groups.

Reply: We sincerely apologize for this oversight. We have carefully verified the accuracy of all figures, and the figure legends have been revised accordingly.

11. Revise the Data Availability statement to comply with PLOS ONE requirements for access to the underlying experimental and proteomic data.

Reply: Thank you for your suggestion. We have revised the “Data Availability Statement” to fully comply with the “Data Availability Policy” of PLOS ONE.

Attachment Submitted filename: Response to Reviewers.doc

# Peer review: Decision Letter 1

**DOI:** 10.1371/journal.pone.0361186.r003

**Authors:** Ryota Tamura

7 Oct 2026

Study on the Molecular Mechanism of Chlorotoxin from Buthus martensii Karsch in the Inhibition of Glioma Angiogenesis

PONE-D-26-32891R1

Dear Dr. Hu,

We’re pleased to inform you that your manuscript has been judged scientifically suitable for publication and will be formally accepted for publication once it meets all outstanding technical requirements.

Within one week, you’ll receive an e-mail detailing the required amendments. When these have been addressed, you’ll receive a formal acceptance letter and your manuscript will be scheduled for publication.

An invoice will be generated when your article is formally accepted. Please note, if your institution has a publishing partnership with PLOS and your article meets the relevant criteria, all or part of your publication costs will be covered. Please make sure your user information is up-to-date by logging into Editorial Manager at Editorial Manager® and clicking the ‘Update My Information' link at the top of the page. For questions related to billing, please contact billing support.

If your institution or institutions have a press office, please notify them about your upcoming paper to help maximize its impact. If they’ll be preparing press materials, please inform our press team as soon as possible -- no later than 48 hours after receiving the formal acceptance. Your manuscript will remain under strict press embargo until 2 pm Eastern Time on the date of publication. For more information, please contact onepress@plos.org.

Kind regards,

Ryota Tamura

Academic Editor

PLOS One

Additional Editor Comments (optional):

Reviewers' comments:

Reviewer's Responses to Questions

**Comments to the Author**

Reviewer #1: All comments have been addressed

**********

2. Is the manuscript technically sound, and do the data support the conclusions?

Reviewer #1: Yes

**********

3. Has the statistical analysis been performed appropriately and rigorously?

Reviewer #1: Yes

**********

4. Have the authors made all data underlying the findings in their manuscript fully available?

The PLOS Data policy

Reviewer #1: Yes

**********

5. Is the manuscript presented in an intelligible fashion and written in standard English?

Reviewer #1: Yes

**********

Reviewer #1: (No Response)

**********

what does this mean?). If published, this will include your full peer review and any attached files.

If you choose “no”, your identity will remain anonymous but your review may still be made public.

**Do you want your identity to be public for this peer review?**  For information about this choice, including consent withdrawal, please see our Privacy Policy

Reviewer #1: No

**********

# Editor report: Acceptance letter

**DOI:** 10.1371/journal.pone.0361186.r004

**Authors:** Ryota Tamura

PONE-D-26-32891R1

PLOS One

Dear Dr. Hu,

I'm pleased to inform you that your manuscript has been deemed suitable for publication in PLOS One. Congratulations! Your manuscript is now being handed over to our production team.

At this stage, our production department will prepare your paper for publication. This includes ensuring the following:

* All references, tables, and figures are properly cited

* All relevant supporting information is included in the manuscript submission,

* There are no issues that prevent the paper from being properly typeset

You will receive further instructions from the production team, including instructions on how to review your proof when it is ready. Please keep in mind that we are working through a large volume of accepted articles, so please give us a few days to review your paper and let you know the next and final steps.

Lastly, if your institution or institutions have a press office, please let them know about your upcoming paper now to help maximize its impact. If they'll be preparing press materials, please inform our press team within the next 48 hours. Your manuscript will remain under strict press embargo until 2 pm Eastern Time on the date of publication. For more information, please contact onepress@plos.org.

You will receive an invoice from PLOS for your publication fee after your manuscript has reached the completed accept phase. If you receive an email requesting payment before acceptance or for any other service, this may be a phishing scheme. Learn how to identify phishing emails and protect your accounts at https://explore.plos.org/phishing.

If we can help with anything else, please email us at customercare@plos.org.

Thank you for submitting your work to PLOS One and supporting open access.

Kind regards,

PLOS One Editorial Office Staff

on behalf of

Dr. Ryota Tamura

Academic Editor

PLOS One
